# Development and validation of a diagnostic nomogram integrating anatomical scores and systemic immune-inflammatory biomarkers for *De Novo* metastatic renal cell carcinoma: a single-center, retrospective study (2016–2025)

**DOI:** 10.3389/fimmu.2026.1747057

**Published:** 2026-03-19

**Authors:** Xu Chen, Yucheng Liu, Tianyu Wang, Disheng Liu, Pengcheng Chang, Nianjun Liu, Weiping Li, Suoshi Jing

**Affiliations:** 1 The First School of Clinical Medicine, Lanzhou University, Lanzhou, China; 2 Department of Urology, the First Hospital of Lanzhou University, Lanzhou, China; 3 Department of Radiology, the First Hospital of Lanzhou University, Lanzhou, China

**Keywords:** machine learning, metastasis, nomograms, Padua score, renal cell carcinoma, systemic immune-inflammatory biomarkers

## Abstract

**Background:**

At initial diagnosis, approximately 30% of renal cell carcinoma (RCC) patients have *de novo* metastasis. This study aims to develop and validate a diagnostic nomogram for predicting cancer metastasis in patients with initially diagnosed RCC.

**Methods:**

A retrospective analysis was conducted in accordance with the TRIPOD (Transparent Reporting of a Multivariable Prediction Model for Individual Prognosis or Diagnosis) reporting guideline, involving patients with RCC treated at the First Hospital of Lanzhou University from January 2016 to August 2025. Patients were randomly assigned to a training cohort and a validation cohort in a 7:3 ratio. Variable selection was performed using three machine learning algorithms: LASSO, SVM-RFE, and Boruta. Independent predictors were identified through multivariate logistic regression, and a diagnostic nomogram was constructed. Model performance was evaluated using receiver operating characteristic (ROC) curves, area under the curve (AUC), calibration curves, and decision curve analysis (DCA).

**Results:**

Three independent predictors were identified: lymphocyte-to-monocyte ratio (LMR) (OR = 0.78, 95% CI: 0.62-0.98), serum albumin-to-alkaline phosphatase ratio (AAPR) (OR = 0.05, 95% CI: 0.01–0.36), and PADUA (Preoperative Aspects and Dimensions Used for an Anatomical) score (OR = 1.41, 95% CI: 1.18–1.69). The AUC of the nomogram was 0.771 in the training cohort and 0.747 in the validation cohort. Calibration curves demonstrated excellent agreement between predicted and actual probabilities, while decision curve analysis highlighted the nomogram’s net clinical benefit across a wide range of threshold probabilities.

**Conclusion:**

The developed nomogram demonstrated moderate discriminatory ability and high clinical applicability in identifying cancer metastasis in patients with initially diagnosed RCC. However, further validation with larger sample sizes and multicenter external cohorts is essential to confirm its generalizability.

## Introduction

As the predominant pathological type of malignant kidney tumor, renal cell carcinoma (RCC) represents a substantial global disease burden. Epidemiological data indicate that kidney cancer ranks ninth in incidence among male malignancies and fifteenth among females (1). In 2024, over 420,000 new cases of RCC were diagnosed globally (2). With the advent of an aging society, the incidence rate of RCC has been gradually increasing worldwide (3). Smoking, obesity, hypertension, and chronic kidney disease are currently recognized as major risk factors (4). RCC often presents insidiously, with only a subset of patients exhibiting typical clinical symptoms (gross hematuria, flank pain, palpable abdominal mass) (5). Most cases are detected incidentally during imaging examinations for other reasons (2).

For patients with non-metastatic RCC, surgery remains the standard of care (6). Specifically, for those with tumors smaller than 4 cm, partial nephrectomy is associated with a 5-year cancer-specific survival rate exceeding 94% (2). In contrast, patients diagnosed with *de novo* metastatic RCC (mRCC) are typically managed with first-line combination therapies incorporating tyrosine kinase inhibitors and immune checkpoint inhibitors (7). Nonetheless, owing to limitations in early diagnostic capabilities (8), approximately 30% of RCC patients are initially diagnosed with regional or distant metastatic disease (9). The most common sites of metastasis for RCC include the lungs, followed by the bones, liver, and brain (2). The prognosis for these mRCC patients is generally poor, with a 5-year survival rate of merely 15% (10). Thus, the early detection of mRCC is essential to improving outcomes.

Previous studies have explored risk factors associated with *de novo* metastasis in RCC. However, the majority have focused on single categories of variables, such as pathological features or anatomical parameters (11, 12). By contrast, there has been limited inclusion of readily available clinical variables, including systemic immune-inflammatory biomarkers and hematological indices. Moreover, there is a paucity of research that systematically integrates and compares multiple indicators to identify the optimal combination of predictive factors. Therefore, there is an urgent clinical need for an individualized metastasis risk assessment tool that incorporates key indicators and is user-friendly for clinicians, thereby facilitating treatment decisions at initial diagnosis and the formulation of follow-up strategies.

Several standardized anatomical classification scoring systems, such as the R.E.N.A.L. (Radius, Exophytic/endophytic properties, Nearness of tumor deepest portion to the collecting system or sinus, Anterior/posterior, Location), PADUA (Preoperative Aspects and Dimensions Used for an Anatomical) scores and renal tumor contact surface area (CSA), have been proposed to optimize nephrectomy outcomes (13). These scoring systems are predominantly employed to assess surgical complexity and predict postoperative complications. In recent years, the field of cancer biology has transitioned from a “cancer cell-centric” paradigm to a broader perspective that positions cancer cells within a dynamic network of stromal cells, encompassing fibroblasts, inflammatory immune cells, and vascular cells collectively referred to as the tumor microenvironment (TME). Within this context, inflammatory responses exert a profound influence on the TME, modulating critical malignant processes, including tumor proliferation, angiogenesis, invasion, and metastasis (14). As pivotal elements of the immune response, peripheral blood immune cells and their derived ratios—notably the neutrophil-to-lymphocyte ratio (NLR) and the lymphocyte-to-monocyte ratio (LMR)—have been validated as robust indicators of both the tumor immune microenvironment and systemic inflammation. These biomarkers have proven instrumental in evaluating tumor aggressiveness and forecasting patient prognosis across various cancers (15–17). Consequently, in the present study, we innovatively incorporated anatomical scoring systems and systemic immune-inflammatory biomarkers into our analytical framework to identify the optimal combination of predictive factors and enhance predictive accuracy.

This study aims to develop and validate a diagnostic nomogram for assessing metastatic risk in patients with initially diagnosed RCC. By employing rigorous feature selection, readily available clinical variables were incorporated to ensure the model’s high applicability and practical utility in routine clinical practice.

## Patients and methods

### Study participants

This study was approved by the Medical Ethics Committee of the First Affiliated Hospital of Lanzhou University (Approval No. LDYYLL-2025-2032). The research strictly adhered to the principles of the Declaration of Helsinki.

We performed a retrospective review of a total of 568 patients who were initially diagnosed with renal malignant tumors between January 2016 and August 2025 at the First Affiliated Hospital of Lanzhou University. Patients with pathologically confirmed RCC and definitive pathological or imaging data at initial diagnosis to determine metastatic status were included. Additionally, patients with other malignant tumors or missing more than 20% data for candidate variables were excluded. This study followed the TRIPOD (Transparent Reporting of a Multivariable Prediction Model for Individual Prognosis or Diagnosis) reporting guideline.

### Outcome definition

We define *de novo* mRCC as any distant organ metastasis confirmed by imaging or pathology reports within a 15-day window before and a 30-day window after the index date of RCC. The index date refers to the date of the first imaging examination that was highly suggestive of RCC. Evidence of distant metastasis is derived from examinations such as chest computed tomography, abdominal and pelvic computed tomography, bone scintigraphy, or head magnetic resonance imaging, explicitly described in radiology reports, or confirmed by pathological examination of metastatic lesions via biopsy. This time window is established based on clinical pathway rationale: some patients primarily seek medical attention for symptoms arising from metastatic lesions (18, 19). Once metastatic lesions are detected on imaging, clinicians typically conduct a prompt search for the primary tumor (confirmed within approximately 15 days). Similarly, the 30-day grace period following diagnosis ensures that the initial imaging evaluation is fully completed.

### Candidate variables

Data for this study were retrospectively collected from the hospital’s comprehensive medical record system, including (1): demographic and tumor characteristics such as age, gender, body mass index (BMI), marital status, smoking history, hypertension, diabetes, tumor pathology types, and tumor laterality (2); laboratory parameters derived from the patient’s initial admission examination, encompassing baseline complete blood count, biochemical indicators, as well as urine protein and occult blood tests; Additionally (3), tumor anatomical complexity was assessed by calculating the PADUA score and CSA from the initial abdominal computed tomography. All assessments were performed independently by two physicians. Any discrepancies were resolved through consensus discussions. Finally (4), a panel of systemic immune-inflammatory markers was computed from baseline laboratory data, including the LMR, NLR, prognostic nutritional index (PNI), derived neutrophil-to-lymphocyte ratio (dNLR), platelet-to-lymphocyte ratio (PLR), systemic immune-inflammation index (SII), systemic inflammatory response index (SIRI), platelet-to-neutrophil ratio (PNR), and albumin-to-alkaline phosphatase ratio (AAPR), the specific calculation formulas for which are provided in **Supplementary Table 1**. All data were entered into EpiData software (version 3.1) using a double-entry method, with consistency verified by cross-checking between the two datasets. Any discrepancies identified were reconciled with the original medical records for verification and subsequent correction.

### Statistical analysis

All statistical analyses were performed using R software (version 4.4.1). Missing values were handled using multiple imputation with the “mice” R package. The distribution of continuous variables was assessed with the Shapiro-Wilk test. Normally distributed data were presented as mean ± standard deviation and compared using independent samples t-tests (for two groups) or one-way ANOVA (for multiple groups); variables with skewed distributions were expressed as median (interquartile range) and compared using the Wilcoxon rank-sum test (for two groups) or the Kruskal-Wallis test (for multiple groups). Levene’s test verified homogeneity of variance. Categorical variables were summarized as frequency (percentage) and compared using the chi-square or Fisher’s exact test, as appropriate.

Variables with a univariate association of P < 0.05 were subsequently subjected to feature selection using three machine learning algorithms: LASSO, SVM-RFE, and Boruta. Features consistently identified by all three methods were retained as candidate predictors and included in a multivariate logistic regression model to identify independent predictors. Restricted Cubic Spline (RCS) analysis was employed to evaluate the dose-response relationship between independent predictors and the outcome. Based on the final model, a nomogram was constructed using the “rms” R package.

Model discrimination was assessed using the area under the receiver operating characteristic curve (AUC); calibration was evaluated using the Hosmer-Lemeshow test and calibration curves; and clinical utility was analyzed via decision curve analysis (DCA). All statistical tests were two-sided, with P < 0.05 considered statistically significant.

## Results

### Baseline patient characteristics

**Figure 1:**
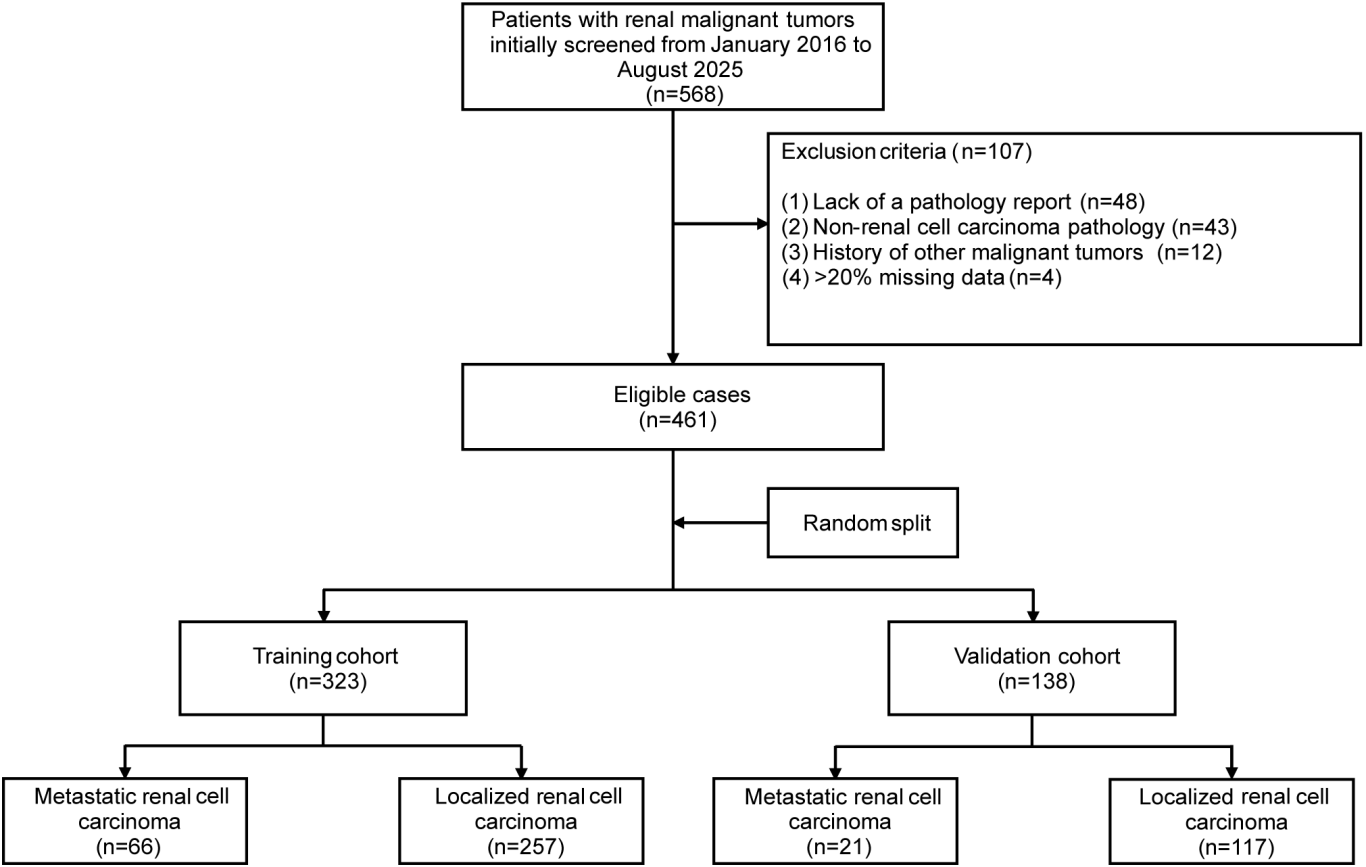
Flowchart of participant inclusion.

This study included 461 patients with RCC (**Figure 1**). The mean age at diagnosis was 61.18 years, and most patients were male (65.2%). Histologically, clear cell RCC was the most prevalent (397/461, 86.1%), followed by chromophobe RCC (30/461, 6.5%). At initial diagnosis, distant metastases were identified in 87 patients (18.9%), comprising 66 cases with single-organ involvement and 21 cases with multi-organ involvement. Among those with single-organ metastasis, the lung was the predominant metastatic site (31/66, 47.0%), followed by bone (15/66, 22.8%), liver (11/66, 16.7%), and other organs (8/66, 12.1%). All patients were randomly assigned in a 7:3 ratio to the training cohort (n=323) and internal validation cohort (n=138) (20). Comparisons of baseline characteristics between the training and validation cohorts are detailed in **Supplementary Table 2**. No significant differences in age, sex, marital status, pathological subtype, or *de novo* metastatic status were observed between the two cohorts (all P > 0.05), indicating successful randomization and that the validation cohort adequately represented the overall study population.

### Univariate analysis

Univariate analysis revealed that *de novo* mRCC was significantly associated with multiple factors (**Table 1**). In the training cohort, no significant differences were observed between the localized RCC and the mRCC groups in terms of diagnostic age, gender, marital status, smoking history, diabetes history, and pathological type (all P > 0.05). However, patients in the mRCC group demonstrated a significantly lower BMI compared to those in the localized RCC group (23.93 vs. 22.17, P < 0.001).

**Table 1:** Comparison of baseline characteristics between localized and *de novo* mRCC patients in the training cohort.

Variable	Localized RCC (*N=257*)	*De novo* mRCC (*N=66*)	*P*-value
Age at Diagnosis(years)	61.05 ± 11.97	62.48 ± 10.94	0.379
Gender:			0.151
Male	162 (63.04%)	48 (72.73%)	
Female	95 (36.96%)	18 (27.27%)	
BMI(kg/m^2^)	23.93 [22.04;26.22]	22.17 [19.37;25.09]	<0.001
Marital Status:			>0.999
Married	249 (96.89%)	65 (98.48%)	
Unmarried	1 (0.39%)	0 (0.00%)	
Widowed	7 (2.72%)	1 (1.52%)	
Smoking:			0.346
No	219 (85.21%)	53 (80.30%)	
Yes	38 (14.79%)	13 (19.70%)	
Hypertension:			0.011
No	167 (64.98%)	54 (81.82%)	
Yes	90 (35.02%)	12 (18.18%)	
Diabetes:			0.292
No	225 (87.55%)	61 (92.42%)	
Yes	32 (12.45%)	5 (7.58%)	
WBC Count(×10^9^ cells/L)	5.86 [4.85;7.00]	6.27 [5.17;7.96]	0.026
RBC(×10^12^ cells/L)	4.77 [4.43;5.08]	4.55 [4.14;4.95]	0.013
HGB(g/L)	146.00 [134.00;158.00]	133.50 [115.25;149.00]	<0.001
HCT(%)	43.90 [40.90;47.40]	41.00 [36.58;45.48]	<0.001
Platelet Count(×10^9^cells/L)	201.00 [164.00;245.00]	227.50 [193.25;292.00]	<0.001
Lymphocyte Count(×10^9^cells/L)	1.50 [1.20;1.88]	1.33 [1.15;1.60]	0.008
Lymphocyte(%)	27.22 [21.09;33.16]	21.45 [15.61;27.70]	<0.001
Monocyte Count(×10^9^cells/L)	0.35 [0.28;0.44]	0.46 [0.33;0.60]	<0.001
Monocyte(%)	6.05 [5.17;7.37]	7.01 [5.89;8.90]	<0.001
Neutrophil Count(×10^9^cells/L)	3.56 [2.82;4.77]	4.30 [3.31;5.76]	0.002
Neutrophil(%)	63.94 [57.41;70.78]	68.53 [61.60;74.18]	0.001
Eosinophil Count(×10^9^cells/L)	0.09 [0.05;0.15]	0.08 [0.04;0.15]	0.764
Basophil Count(×10^9^cells/L)	0.02 [0.01;0.03]	0.02 [0.01;0.03]	0.181
AST(U/L)	21.00 [17.00;26.00]	20.00 [15.25;24.75]	0.221
ALT(U/L)	21.00 [15.00;31.00]	18.00 [14.00;23.75]	0.037
TP(g/L)	70.90 [66.30;75.60]	71.40 [67.40;75.20]	0.642
ALB(g/L)	43.80 [40.90;45.90]	41.75 [37.82;44.20]	<0.001
GLB(g/L)	27.40 [24.60;30.40]	29.70 [27.00;33.05]	<0.001
ALP(U/L)	84.00 [68.50;101.00]	95.75 [82.93;115.38]	<0.001
GGT(U/L)	25.60 [17.80;43.20]	27.20 [17.50;49.33]	0.287
CHE(KU/L)	8.04 [6.87;9.31]	6.78 [5.90;8.36]	<0.001
AFU(U/L)	19.00 [15.00;24.00]	18.00 [15.00;22.00]	0.409
Urea(mmol/L)	5.69 [4.68;6.84]	5.86 [4.80;7.20]	0.593
Cr(μmol/L)	72.00 [62.00;84.00]	72.95 [64.62;85.67]	0.639
UA(μmol/L)	326.00 [271.00;375.00]	307.50 [259.25;359.50]	0.179
Calcium(mmol/L)	2.22 [2.13;2.31]	2.20 [2.11;2.30]	0.429
P(mmol/L)	1.11 ± 0.18	1.11 ± 0.17	0.996
Glu(mmol/L)	5.00 [4.52;5.70]	5.18 [4.68;6.06]	0.242
TG(mmol/L)	1.25 [0.95;1.82]	1.02 [0.81;1.36]	0.012
TC(mmol/L)	4.13 [3.44;4.76]	3.79 [3.20;4.53]	0.047
Proteinuria:			0.587
-	205 (79.77%)	50 (75.76%)	
+	47 (18.29%)	13 (19.70%)	
++	4 (1.56%)	3 (4.55%)	
+++	1 (0.39%)	0 (0.00%)	
Occult Blood:			0.002
-	179 (69.65%)	32 (48.48%)	
+	78 (30.35%)	34 (51.52%)	
PNI	51.30 [47.85;54.90]	48.78 [45.52;51.80]	<0.001
NLR	2.28 [1.73;3.31]	3.14 [2.29;4.71]	<0.001
dNLR	1.77 [1.35;2.42]	2.14 [1.58;2.81]	0.004
LMR	4.38 [3.31;5.57]	2.89 [2.32;4.63]	<0.001
PLR	125.90 [97.74;166.47]	179.26 [125.57;254.29]	<0.001
SII(10^9^cells/L)	461.67 [308.96;712.53]	734.26 [483.32;1224.21]	<0.001
SIRI(10^9^cells/L)	0.81 [0.54;1.25]	1.44 [0.81;2.34]	<0.001
PNR	54.83 [40.93;70.83]	54.19 [41.13;77.69]	0.499
PMR	550.00 [420.00;733.33]	518.64 [391.27;765.08]	0.571
AAPR	0.52 [0.43;0.65]	0.44 [0.36;0.52]	<0.001
Corrected Calcium(mmol/L)	2.15 [2.08;2.22]	2.15 [2.10;2.21]	0.278
Laterality:			0.470
Left	142 (55.25%)	32 (48.48%)	
Right	114 (44.36%)	34 (51.52%)	
Both	1 (0.39%)	0 (0.00%)	
Pathology:			0.439
ccRCC	221 (85.99%)	57 (86.36%)	
pRCC	9 (3.50%)	4 (6.06%)	
chRCC	19 (7.39%)	2 (3.03%)	
Other	8 (3.11%)	3 (4.55%)	
PADUA score	9.00 [8.00;11.00]	11.00 [9.00;12.00]	<0.001
CSA(cm^2^)	28.46 [16.74;57.77]	72.48 [39.07;115.09]	<0.001

AAPR, albumin-to-alkaline phosphatase ratio; AFU,α-L-fucosidase; ALB, albumin; ALP, alkaline phosphatase; ALT, alanine aminotransferase; AST, aspartate aminotransferase; BMI, body mass index; ccRCC, clear cell renal cell carcinoma; CHE, cholinesterase; chRCC, chromophobe renal cell carcinoma; Cr, creatinine; CSA, contact surface area; dNLR, derived neutrophil-to-lymphocyte ratio; GGT, γ-glutamyl transferase; GLB, globulin; Glu, glucose; HCT, hematocrit; HGB, hemoglobin; LMR, lymphocyte-to-monocyte ratio; NLR, neutrophil-to-lymphocyte ratio; P, phosphorus; PADUA score, Preoperative Aspects and Dimensions Used for an Anatomical; PLR, platelet-to-lymphocyte ratio; PMR, platelet-to-monocyte ratio; PNI, prognostic nutritional index; PNR, platelet-to-neutrophil ratio; pRCC, papillary renal cell carcinoma; RBC, red blood cell; SII, systemic immune-inflammation index; SIRI, systemic inflammatory response index; carcinoma; TC, total cholesterol; TG, triglycerides; TP, total protein; UA, uric acid; WBC, white blood cell.

In terms of laboratory parameters, the mRCC group demonstrated elevated white blood cell counts and platelet counts, but significantly lower red blood cell counts, hemoglobin concentration, and hematocrit. Notably, despite elevated white blood cell counts in the mRCC group, both the absolute lymphocyte count and lymphocyte percentage were significantly lower than in the localized RCC group (1.50 vs. 1.33, P = 0.008; 27.22% vs. 21.45%, P < 0.001). Additionally, patients in the mRCC group exhibited lower serum albumin (ALB) levels and higher globulin levels, suggesting poorer nutritional status and heightened immune activation.

Regarding systemic immune-inflammatory markers, significant differences were observed between the two groups for the following parameters: PNI, NLR, dNLR, LMR, PLR, SII, SIRI, and AAPR (all P < 0.05),. However, PNR and PMR did not show statistically significant differences. In terms of tumor anatomical characteristics, the mRCC group exhibited a significantly higher PADUA score and CSA compared to the localized RCC group (PADUA score: 9 vs. 11, P < 0.001; CSA: 28.46 cm² vs. 72.48 cm², P < 0.001).

### Machine learning

**Figure 2:**
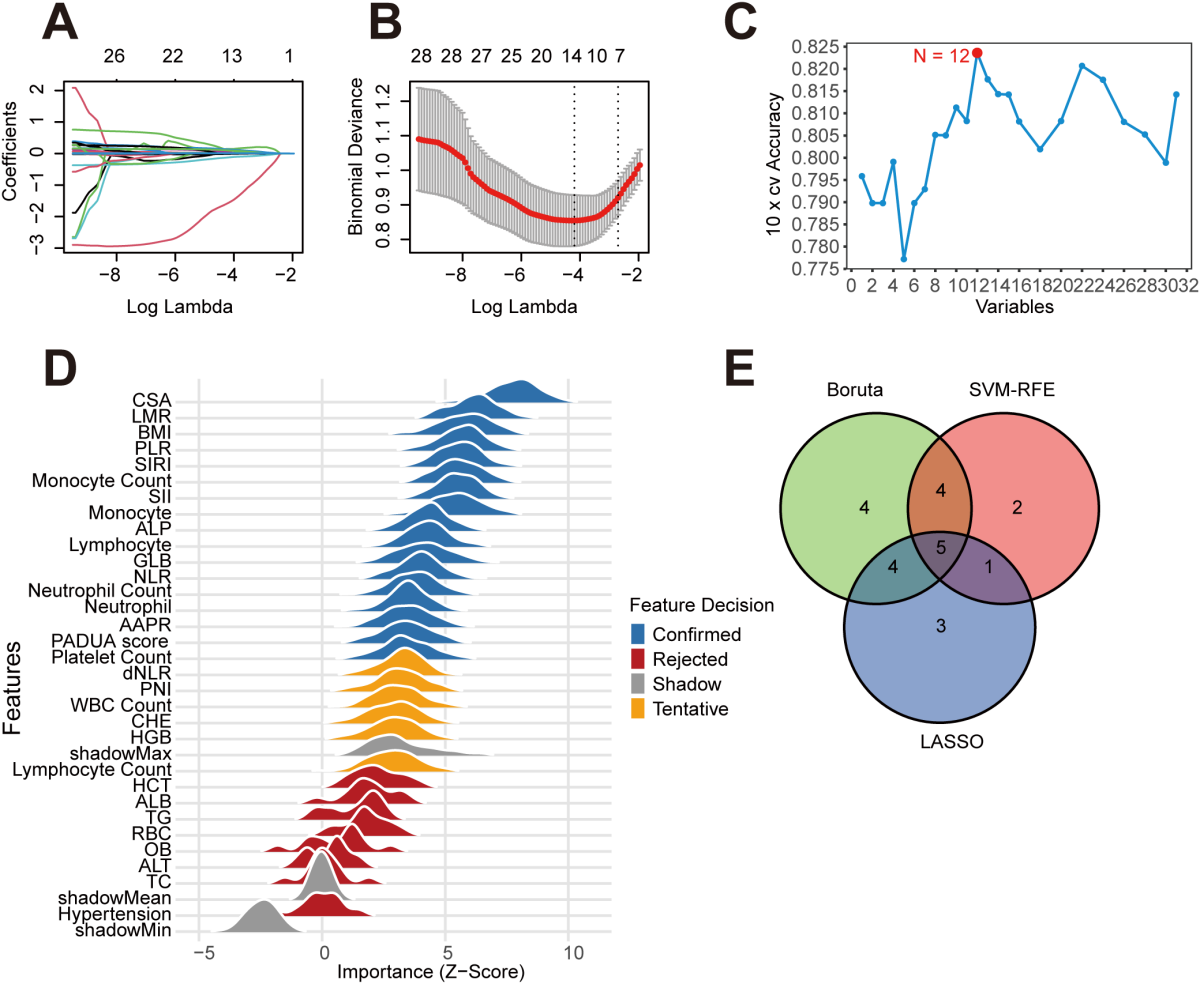
Optimize the screening variables using LASSO, SVM-RFE, and Boruta. **(A)** LASSO coefficient path diagram. **(B)** LASSO cross-validation curve to identify the optimal lambda through 10-fold cross-validation. **(C)** Evaluation of model accuracy under different feature counts using the SVM-RFE algorithm with 10-fold cross-validation. **(D)** The Boruta algorithm evaluates the importance distribution of each feature through ridge plots. Features in the Confirmed state are retained due to their significant association with the outcome variable. **(E)** The Venn diagram illustrates the intersection of variables selected by three feature selection methods. The overlapping region comprises five jointly selected biomarkers: LMR, AAPR, Monocyte Count, SIRI, and PADUA score. AAPR, albumin-to-alkaline phosphatase ratio; LMR, lymphocyte-to-monocyte ratio; PADUA score, Preoperative Aspects and Dimensions Used for an Anatomical; SIRI, systemic inflammatory response index.

The 31 statistically significant predictors from univariate analysis were incorporated into three machine learning methods for further feature selection (**Figure 2**). LASSO regression was performed using the “glmnet” R package, with the optimal penalty coefficient λ determined via 10-fold cross-validation. The lambda.min value was selected as the final criterion, ultimately identifying 13 predictive factors. For the SVM-RFE method, 10-fold cross-validation was also employed, using a radial basis function SVM as the classifier for recursive feature elimination. The model’s classification accuracy for each feature subset was evaluated through resampling, which determined that the highest accuracy was achieved with 12 variables. Further application of the Boruta algorithm, with 200 iterations, confirmed 17 variables as significantly associated with *de novo* mRCC. Intersection analysis of the screening results from the three methods ultimately identified five common predictors (monocyte count, LMR, SIRI, AAPR, PADUA score) for subsequent in-depth analysis.

### Multivariate analysis and nomogram development

**Figure 3:**
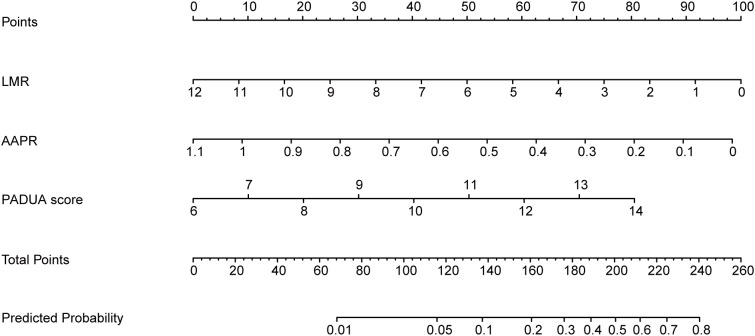
A nomogram model for predicting the risk of metastasis at initial diagnosis in RCC patients. By connecting corresponding points for each variable, a total score is obtained, which, in turn, estimates an individual’s probability of metastasis.

Multivariate logistic regression analysis identified LMR, AAPR, and PADUA score as independent predictors of *de novo* mRCC (all P < 0.05). However, after multivariate adjustment, monocyte count and SIRI showed no significant association with mRCC (all P > 0.05) (**Table 2**). The multicollinearity test confirmed that the variance inflation factor (VIF) for all variables was below 5 (**Supplementary Table 3**), indicating no severe multicollinearity in the model. Based on the multivariate analysis results, we selected LMR, AAPR, and PADUA score to construct a clinical prediction nomogram for clinicians as a convenient risk assessment tool (**Figure 3**).

**Table 2:** Multivariate logistic analysis for predictors of *de novo* mRCC.

Variable	Beta	SE	OR (95% CI)	*P*-value
Monocyte Count (×10^9^cells/L)	1.087	1.025	2.96 (0.40-22.09)	0.289
LMR	-0.251	0.117	0.78 (0.62-0.98)	0.032
SIRI (×10^9^cells/L)	-0.041	0.077	0.96 (0.83-1.12)	0.595
AAPR	-2.982	1.004	0.05 (0.01-0.36)	0.003
PADUA score	0.346	0.092	1.41 (1.18-1.69)	<0.001

AAPR, albumin-to-alkaline phosphatase ratio; LMR, lymphocyte-to-monocyte ratio; PADUA score, Preoperative Aspects and Dimensions Used for an Anatomical; SIRI, systemic inflammatory response index.

### Dose-response analysis

**Figure 4:**
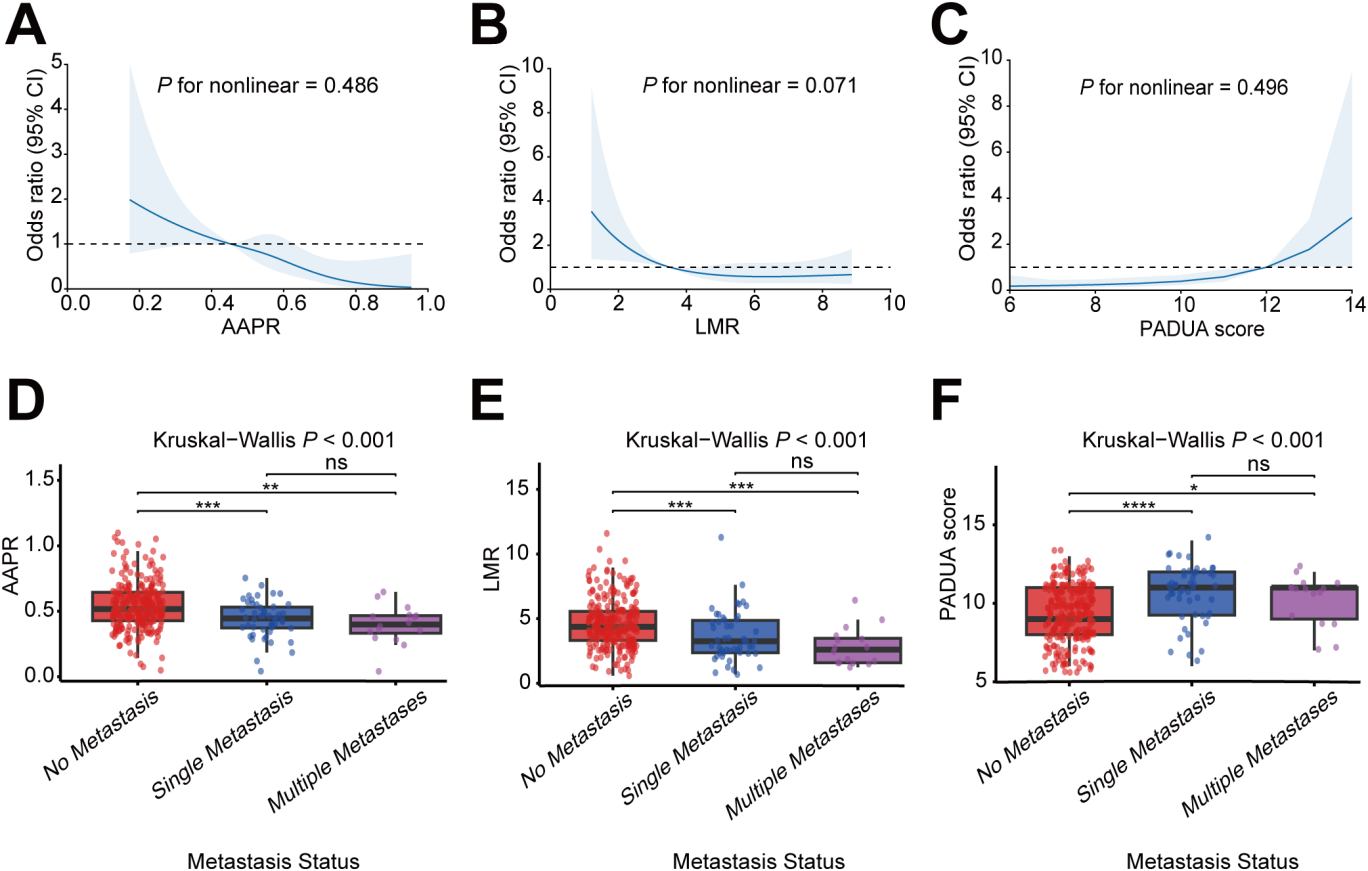
Dose–response associations of predictors and *de novo* mRCC. **(A–C)** Restricted Cubic Spline models demonstrate the dose-response relationship between **(A)** AAPR, **(B)** LMR, and **(C)** PADUA score and the risk of metastasis in initially diagnosed RCC. Solid lines represent adjusted odds ratios, shaded areas denote 95% confidence intervals, and dashed lines indicate the reference value (OR = 1). **(D–F)** Box plots illustrate the distributions of **(D)** AAPR, **(E)** LMR, and **(F)** PADUA score among patients with no metastasis, single-organ metastasis, and multiple-organ metastasis. Asterisks denote statistical significance in pairwise comparisons between groups (*P < 0.05, **P < 0.01, ***P < 0.001). AAPR, albumin-to-alkaline phosphatase ratio; LMR, lymphocyte-to-monocyte ratio; PADUA score, Preoperative Aspects and Dimensions Used for an Anatomical.

Based on the restricted cubic spline analysis, we conducted an in-depth investigation into the association between AAPR, LMR, and PADUA score and *de novo* mRCC. This analysis utilized the “plotRCS” package, setting nodes at the 0.05th, 0.35th, 0.65th, and 0.95th percentiles. Results demonstrated that AAPR, LMR, and PADUA score all exhibited linear associations with *de novo* mRCC (P for nonlinear: 0.486, 0.071, and 0.496, respectively) (**Figure 4**). The risk of distant metastasis at initial diagnosis progressively increased with decreasing AAPR and LMR and with increasing PADUA score. Compared to the localized RCC group, the single-organ metastasis group had significantly lower AAPR and LMR and a considerably higher PADUA score (all P < 0.001). However, no statistically significant differences were observed for these three indicators between the single-organ metastasis and multi-organ metastasis groups.

### ROC analysis, calibration curve, and clinical decision curve analysis

**Figure 5:**
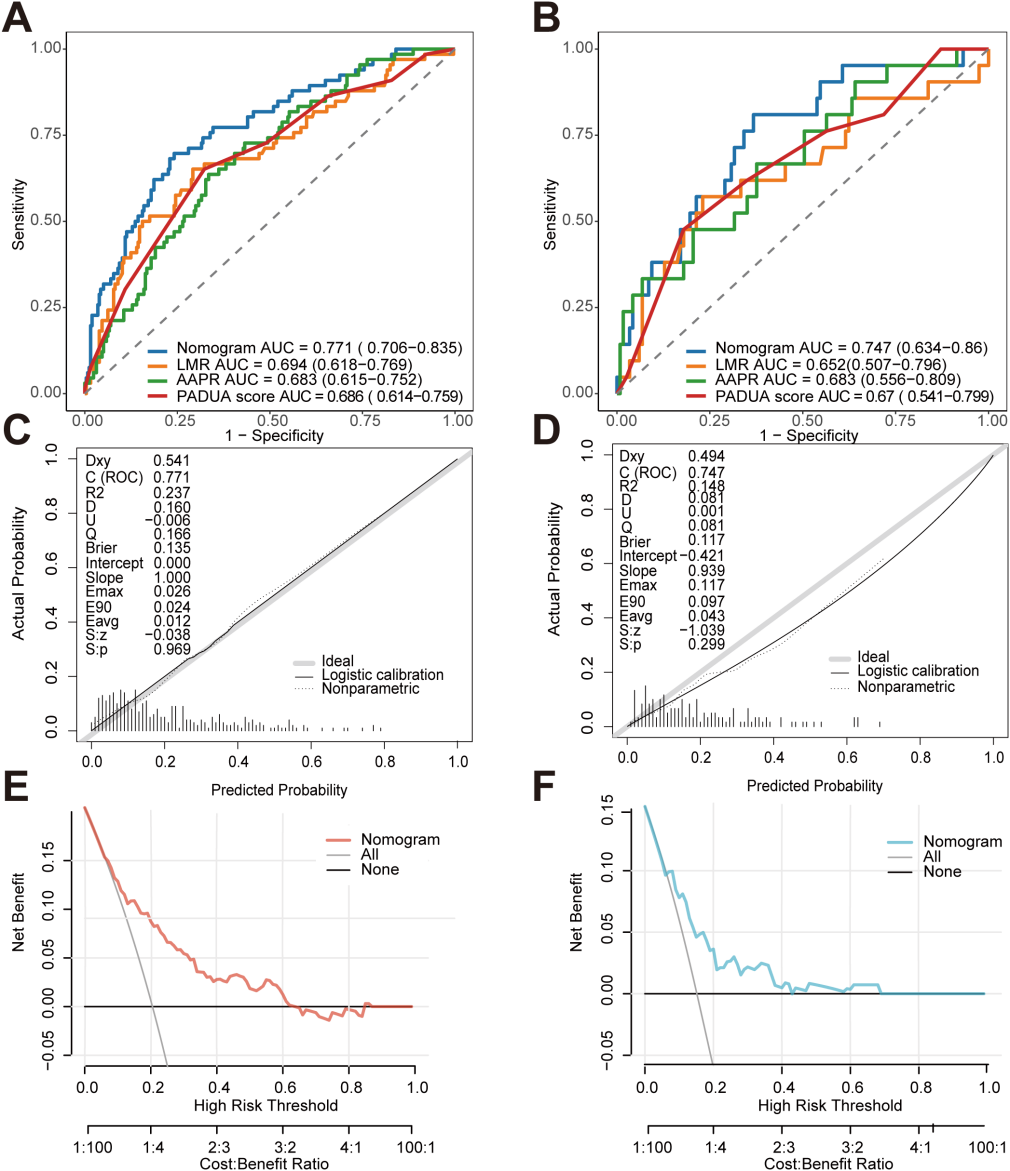
Evaluating model performance on the training and validation cohorts. **(A, B)** Comparison of receiver operating characteristic curves between the nomogram model and single predictors in **(A)** the training cohort and **(B)** the validation cohort. The legend shows the area under the curve (AUC) and 95% confidence intervals for each model. **(C, D)** Calibration curves for model prediction probabilities versus actual observed probabilities in **(C)** the training cohort and **(D)** the validation cohort. The diagonal line represents ideal calibration, the solid curve shows the model’s actual calibration, and the dashed line indicates the calibration curve after logistic correction. **(E, F)** Decision curve analysis of the nomogram model in **(E)** the training cohort and **(F)** the validation cohort. The curves illustrate the net benefit of using this model at different decision thresholds, compared against the “treat all” and “treat none” strategies.

The predictive performance of the model was systematically evaluated using ROC curves, calibration curves, and decision curve analysis (**Figure 5**). ROC analysis revealed that the nomogram demonstrated good predictive accuracy in both the training and validation cohorts, with AUC values of 0.771 (95% CI: 0.706–0.835) and 0.747 (95% CI: 0.634–0.860), respectively. Using the optimal probability threshold derived from the ROC analysis, the model achieved a sensitivity of 63.6% and a specificity of 77.4% in the training cohort, and a sensitivity of 53.1% and a specificity of 77.8% in the validation cohort. Calibration curves assessed via the Hosmer-Lemeshow test demonstrated good consistency between predicted and actual probabilities (training cohort: P = 0.769; validation cohort: P = 0.603). DCA results indicated that, in the training cohort, the clinical net benefit of using the model exceeded that of the “treat all” and “treat none” strategies when the threshold probability ranged from 0% to 63%. In the validation cohort, the model demonstrated superior clinical utility to extreme strategies across two threshold intervals: 0%–42% and 44%–68%.

## Discussion

This study developed and validated a diagnostic nomogram that integrates systemic immune-inflammatory biomarkers with radiographic anatomical scores to predict the risk of distant metastasis in patients with initially diagnosed RCC. The model, presented as a clinically accessible nomogram, demonstrated robust performance upon internal validation, exhibiting moderate discriminatory power, satisfactory calibration, and promising clinical utility.

Compared with previous studies, most current RCC metastasis prediction models are constructed using public databases, with prediction targets often limited to specific metastatic sites (such as lung, liver, or bone) (21–23). Furthermore, these models typically incorporate numerous variables, placing particular emphasis on tumor morphological or pathological features while rarely integrating laboratory indicators and imaging characteristics (21, 22). The model developed in this study incorporates only three core variables (LMR, AAPR, and PADUA score), which comprehensively account for the multidimensional characteristics of RCC while being derived entirely from routine initial diagnostic examinations. Although the model exhibits moderate discriminative power, this approach ensures high clinical applicability and accessibility. Beyond its simplicity, decision curve analysis demonstrated that across a wide range of threshold probabilities in both the training cohort (0%–63%) and the validation cohort (0%–42% and 44%–68%), the net benefit of using our model exceeded that of the “treat all” or “treat none” strategies. This favorable net benefit further supports the model’s potential to assist clinicians in risk stratification and clinical decision-making at initial diagnosis.

Accumulating evidence supports the prognostic value of the LMR in various malignancies. A meta-analysis of 4,908 gastric cancer patients established that a low LMR is significantly associated with reduced overall survival (24). Therefore, a low LMR is more conducive to tumor cell invasion and dissemination. Furthermore, LMR has been shown to correlate with immunotherapy efficacy (22); for example, in a retrospective study of 571 mRCC patients treated with nivolumab, those with a high LMR had superior overall survival (25). In prostate cancer, immune checkpoint molecules such as CD96 and PD-L2 have also been associated with biochemical recurrence and may serve as potential therapeutic targets (26), further highlighting the relevance of immune-related biomarkers across different malignancies. RCC is one of the most vascularized tumors due to mutations in the von Hippel-Lindau gene. After anti-angiogenic therapy with sunitinib or pazopanib, the number of proangiogenic monocyte subsets (VEGFR-1+CD14 and Tie2+CD14) decreases in mRCC patients (27). Specifically, a low LMR indicates a relative decrease in lymphocytes or a relative increase in monocytes. Lymphocytes can induce tumor cell apoptosis and inhibit tumor cell invasion. In contrast, monocytes not only promote tumor progression within the TME but can also be induced to differentiate into tumor-associated macrophages, which promote tumor growth and metastasis (28). Similarly, the AAPR has been demonstrated as a significant marker of tumor progression. Studies indicate that an AAPR ≤ 0.48 is significantly correlated with bone and liver metastases in gastric cancer (29). At the same time, in patients with non-metastatic RCC, a low AAPR similarly predicts poor postoperative prognosis (30). Specifically, ALB, as an indicator of systemic inflammation, suppresses tumor cell proliferation and invasion (26). In contrast, alkaline phosphatase may serve as a potential marker of oxidative stress, thereby enhancing tumor invasiveness (31). Therefore, elevated AAPR may indicate relatively increased ALB levels or decreased alkaline phosphatase levels, thereby suppressing tumor cell proliferation and invasion.

This study further establishes the PADUA score as an independent predictor of distant metastasis at initial diagnosis in RCC patients (OR = 1.41, 95% CI: 1.18–1.69). The score integrates key anatomical parameters, such as tumor diameter, exophytic rate, and relationship with the collecting system, and quantifies tumor complexity and invasive potential more effectively than the traditional T stage or simple tumor size (32). Consistent with this anatomical basis, prior research has validated its utility in predicting surgical outcomes, including thermal ischemia time during partial nephrectomy and postoperative acute kidney injury (33, 34). Moreover, its significant correlation with chronic kidney disease progression following CT-guided microwave ablation further underscores the PADUA score’s broader role in stratifying the risk of disease progression and functional decline in RCC (35). This strong correlation with metastatic risk prompted us to explore the potential mechanisms linking tumor anatomy to metastatic behavior. First, higher PADUA scores are partly attributable to larger tumor diameters. Larger tumors undergo more cell divisions, accumulating a greater number of genetic alterations and thereby increasing the likelihood of acquiring metastatic potential (12). Second, tumors that invade or infiltrate the renal sinus receive higher PADUA scores, and renal sinus involvement is a well-established major risk factor for metastasis in RCC (36).

We employed RCS analysis to examine the dose-response relationship between the predictors and the outcome, which revealed linear associations of LMR, PADUA score, and AAPR with *de novo* mRCC. This provides strong statistical justification for including these variables in their original form in the subsequent multivariate logistic regression model (37). However, no significant differences in these three predictors were observed between the single-organ metastasis group and the multi-organ metastasis group, suggesting that the model can only predict the presence of metastasis but not the extent of metastatic burden.

Several limitations must be acknowledged in this study. Its retrospective nature over a nearly 10-year period may make it susceptible to selection bias and missing data. Additionally, although our training cohort included 257 patients with localized RCC and 66 with metastatic RCC, resulting in an events per variable (EPV) of 13.2 for the five candidate variables entered into the multivariate logistic regression, which exceeds the widely accepted threshold of 10 for low overfitting risk, some risk of overfitting may still exist due to the relatively limited sample size (38). The model’s generalizability therefore requires further validation in larger, multi-center prospective cohorts. We plan to expand the sample size in future studies to further assess the model’s stability and clinical utility.

## Conclusion

This study identified LMR, AAPR, and the PADUA score as significant predictors of metastatic disease at the time of RCC diagnosis. Moreover, we developed a diagnostic nomogram for *de novo* mRCC that demonstrated moderate discriminatory ability and high clinical utility. This model serves as a practical tool to assist clinicians in metastasis detection and improve risk stratification at diagnosis.

## Data Availability

The raw data supporting the conclusions of this article will be made available by the authors, without undue reservation.
