# Supplementary material for: Development and validation of a diagnostic nomogram integrating anatomical scores and systemic immune-inflammatory biomarkers for De Novo metastatic renal cell carcinoma: a single-center, retrospective study (2016–2025)
Source: Front Immunol. 2026 Mar 19;17:1747057. doi: 10.3389/fimmu.2026.1747057 (PMC13044033; doi:10.3389/fimmu.2026.1747057)
Supplement: Supplementary file 1 [file Table1.docx]

***Supplementary Tables***

**Supplementary Table 1. Definition and Calculation Formulas for Variables**

| Variable | Full Name | Calculation Formula |
| --- | --- | --- |
| AAPR | Albumin-to-alkaline phosphatase ratio | Albumin(g/L)/Alkaline phosphatase(U/L) |
| BMI (kg/m²) | Body mass index | Weight(kg)/Height^2^ (m^2^) |
| Corrected Calcium (mmol/L) | Corrected Calcium | Actual measurement of blood calcium(mmol/L) +0.02×[40-ALB(g/L)] |
| CSA (cm²) | Contact surface area | 2π×tumor radius(cm)×tumor spherical crown height(cm) |
| dNLR | Derived neutrophil-to-lymphocyte ratio | Neutrophil count(×10^9^cells/L)/ (WBC count(×10^9^cells/L)-neutrophil count(×10^9^cells/L)) |
| LMR | Lymphocyte-to-monocyte ratio | Lymphocyte count(×10^9^cells/L)/monocyte count(×10^9^cells/L) |
| NLR | Neutrophil-to-lymphocyte ratio | Neutrophil count(×10^9^cells/L)/lymphocyte count(×10^9^cells/L) |
| PLR | Platelet-to-lymphocyte ratio | Platelet count(×10^9^cells/L)/lymphocyte count(×10^9^cells/L) |
| PMR | Platelet-to-monocyte ratio | Platelet count(×10^9^cells/L)/monocyte count(×10^9^cells/L) |
| PNI | Prognostic nutritional index | ALB(g/L) +5×lymphocyte count(×10^9^cells/L) |
| PNR | Platelet-to-neutrophil ratio | Platelet count(×10^9^cells/L)/neutrophil count(×10^9^cells/L) |
| SII (×10^9^cells/L) | Systemic immune-inflammation index | (Platelet count(×10^9^cells/L) ×neutrophil count(×10^9^cells/L))/lymphocyte count(×10^9^cells/L) |
| SIRI (×10^9^ cells/L) | Systemic inflammatory response index | (Neutrophil count(×10^9^cells/L) ×monocyte count(×10^9^cells/L))/lymphocyte count(×10^9^cells/L) |

**Supplementary Table 2.Descriptive characteristics of the overall cohort, training set, and validation set**

| **Variable** | **ALL** | **Training** | **Validation** | 1. **overall** |  |
| --- | --- | --- | --- | --- | --- |
|  | ***N=461*** | ***N=323*** | ***N=138*** |  |  |
| Age at diagnosis (years) | 61.00 [54.00;70.00] | 62.00 [54.00;71.00] | 60.00 [54.00;70.00] | 0.589 |  |
| Gender: |  |  |  | 0.592 |  |
| Male | 304 (65.94%) | 210 (65.02%) | 94 (68.12%) |  |  |
| Female | 157 (34.06%) | 113 (34.98%) | 44 (31.88%) |  |  |
| Height(cm) | 168.00 [162.00;173.00] | 168.00 [162.00;172.00] | 169.00 [160.00;174.00] | 0.557 |  |
| Weight(kg) | 67.00 [59.00;75.00] | 65.00 [59.00;75.00] | 69.50 [60.00;75.00] | 0.153 |  |
| BMI(kg/m^2^) | 23.88±3.38 | 23.76±3.39 | 24.15±3.34 | 0.249 |  |
| Marital Status: |  |  |  | 0.087 |  |
| Married | 448 (97.18%) | 314 (97.21%) | 134 (97.10%) |  |  |
| Unmarried | 3 (0.65%) | 1 (0.31%) | 2 (1.45%) |  |  |
| Divorced | 1 (0.22%) | 0 (0.00%) | 1 (0.72%) |  |  |
| Widowed | 9 (1.95%) | 8 (2.48%) | 1 (0.72%) |  |  |
| Smoking: |  |  |  | 0.393 |  |
| No | 383 (83.08%) | 272 (84.21%) | 111 (80.43%) |  |  |
| Yes | 78 (16.92%) | 51 (15.79%) | 27 (19.57%) |  |  |
| Hypertension: |  |  |  | 0.079 |  |
| No | 303 (65.73%) | 221 (68.42%) | 82 (59.42%) |  |  |
| Yes | 158 (34.27%) | 102 (31.58%) | 56 (40.58%) |  |  |
| Diabetes: |  |  |  | 0.451 |  |
| No | 404 (87.64%) | 286 (88.54%) | 118 (85.51%) |  |  |
| Yes | 57 (12.36%) | 37 (11.46%) | 20 (14.49%) |  |  |
| Pathology: |  |  |  | 1.000 |  |
| ccRCC | 397 (86.12%) | 278 (86.07%) | 119 (86.23%) |  |  |
| pRCC | 18 (3.90%) | 13 (4.02%) | 5 (3.62%) |  |  |
| chRCC | 30 (6.51%) | 21 (6.50%) | 9 (6.52%) |  |  |
| Other | 16 (3.47%) | 11 (3.41%) | 5 (3.62%) |  |  |
| Metastasis: |  |  |  | 0.238 |  |
| RCC | 374 (81.13%) | 257 (79.57%) | 117 (84.78%) |  |  |
| mRCC | 87 (18.87%) | 66 (20.43%) | 21 (15.22%) |  |  |

BMI, body mass index; ccRCC, clear cell renal cell carcinoma; chRCC, chromophobe renal cell carcinoma; mRCC, metastatic RCC; pRCC, papillary renal cell carcinoma.

**Supplementary Table 3.** **The variance inflation factor (VIF) test for multivariate logistic regression**

| **Variable** | **VIF** | **Tolerance** |  |
| --- | --- | --- | --- |
|  |  |  |  |
| Monocyte count | 1.782 | 0.561 |  |
| SIRI | 1.630 | 0.613 |  |
| LMR | 1.713 | 0.584 |  |
| AAPR | 1.064 | 0.940 |  |
| PADUA score | 1.003 | 0.997 |  |

AAPR, albumin-to-alkaline phosphatase ratio; LMR, lymphocyte-to-monocyte ratio‌; SIRI, systemic inflammatory response index; PADUA score, Preoperative Aspects and Dimensions Used for an Anatomical.
